# Gene Expression Dynamics in Major Endocrine Regulatory Pathways along the Transition from Solitary to Social Life in a Bumblebee, *Bombus terrestris*

**DOI:** 10.3389/fphys.2016.00574

**Published:** 2016-11-24

**Authors:** Pavel Jedlička, Ulrich R. Ernst, Alena Votavová, Robert Hanus, Irena Valterová

**Affiliations:** ^1^Department of Chemistry of Social Insects, The Institute of Organic Chemistry and Biochemistry, Czech Academy of SciencesPrague, Czechia; ^2^Agricultural Research Ltd.Troubsko, Czechia; ^3^Research Group of Infochemicals, The Institute of Organic Chemistry and Biochemistry, Czech Academy of SciencesPrague, Czechia

**Keywords:** social insects, social evolution, diapause, reproduction, caste differentiation, hormones, endocrine glands

## Abstract

Understanding the social evolution leading to insect eusociality requires, among other, a detailed insight into endocrine regulatory mechanisms that have been co-opted from solitary ancestors to play new roles in the complex life histories of eusocial species. Bumblebees represent well-suited models of a relatively primitive social organization standing on the mid-way to highly advanced eusociality and their queens undergo both, a solitary and a social phase, separated by winter diapause. In the present paper, we characterize the gene expression levels of major endocrine regulatory pathways across tissues, sexes, and life-stages of the buff-tailed bumblebee, *Bombus terrestris*, with special emphasis on critical stages of the queen's transition from solitary to social life. We focused on fundamental genes of three pathways: (1) Forkhead box protein O and insulin/insulin-like signaling, (2) Juvenile hormone (JH) signaling, and (3) Adipokinetic hormone signaling. Virgin queens were distinguished by higher expression of *forkhead box protein O* and downregulated insulin-like peptides and JH signaling, indicated by low expression of *methyl farnesoate epoxidase* (*MFE*) and transcription factor *Krüppel homolog 1* (*Kr-h1*). Diapausing queens showed the expected downregulation of JH signaling in terms of low *MFE* and *vitellogenin* (*Vg*) expressions, but an unexpectedly high expression of *Kr-h1*. By contrast, reproducing queens revealed an upregulation of *MFE* and *Vg* together with insulin signaling. Surprisingly, the insulin growth factor 1 (IGF-1) turned out to be a queen-specific hormone. Workers exhibited an expression pattern of *MFE* and *Vg* similar to that of reproducing queens. Males were characterized by high *Kr-h1* expression and low *Vg* level. The tissue comparison unveiled an unexpected resemblance between the fat body and hypopharyngeal glands across all investigated genes, sexes, and life stages.

## Introduction

The evolution of cooperation, altruism and eusociality is one of the outstanding problems in science (Pennisi, 2005). A huge body of work has been dedicated to the theoretical foundations of eusociality, starting with the classical works of Hamilton (1963). In recent years, genomic studies have helped to address more proximate questions of social evolution (e.g., Toth et al., 2007; Simola et al., 2013; Woodard et al., 2014; Kapheim et al., 2015). However, relatively little is known about the physiology underlying the transitions from the solitary to social life style (Robinson et al., 2005; Woodard et al., 2011), and studies on highly eusocial insects are in some respect of limited value because they represent a highly derived state. In contrast, bumblebees are suitable models in this respect due to their relatively primitive social organization (Amsalem et al., 2015a; Sadd et al., 2015). Within their short lifespan, delimited by the annual life cycle of the colonies in temperate climates, bumblebee queens undergo several dramatic shifts in life style and physiology, starting with a solitary pre-hibernation phase, through solitary diapause and nest founding to the social and reproductive phase as mother queens (e.g., Goulson, 2010). In insects, such transitions are accompanied by significant, hormonally regulated changes in fertility and metabolism (Arrese and Soulages, 2010). Juvenile hormone (JH) is the major regulator of insect development and reproduction (e.g., Riddiford, 2012; Jindra et al., 2013). The metabolism is controlled by two types of neuropeptides and their signaling pathways (Antonova et al., 2012). The first group belongs to the functional homologs of the vertebrate insulin. Insulin-like peptides and insulin/insulin-like growth factors (ILPs/IGFs) make up together a complex signaling network (IIS), which is tightly interconnected with JH signaling (reviewed in e.g., Antonova et al., 2012; Badisco et al., 2013). The second group comprises functionally well described energy mobilizing factors of the adipokinetic hormone family (AKHs), functionally equivalent to the mammalian glucagon (Kim and Rulifson, 2004). Relatively little is known on the integration of these signaling pathways in bumblebees, and especially in the dynamically changing physiology of bumblebee queens, when compared to some models of solitary insects or the socially highly derived European honey bee, *Apis mellifera*.

We selected the *methyl farnesoate epoxidase gene* (*MFE*) as an indicator of JH production, since the expression levels of this gene, coding for the enzyme that catalyzes the final step of JH III biosynthesis, are known to be correlated with JH titers, e.g., in honeybee workers (Bomtorin et al., 2014). MFE was also identified in a number of other insect species (Daimon and Shinoda, 2013), in which, unlike in honeybees, JH preserved its ancestral gonadotropic function reported also in bumblebees (Shpigler et al., 2014, 2016). Further relevant markers studied in both honeybees and bumblebees are the transcription factor Krüppel homolog 1 (Kr-h1) and the yolk protein vitellogenin (Vg). Kr-h1 is the first down-stream transcription factor responding to the activation of JH-specific receptors in developing insects (Jindra et al., 2013). Shpigler et al. (2010, 2014) showed that the ovarian development as well as *Vg* and *Kr-h1* expression are JH-dependent in *B. terrestris* workers and that *Kr-h1* expression is inhibited by the queen's presence. However, Vg's impact on social interactions seems to be much stronger than on the workers' reproductive status and in that case Vg is not regulated by JH signaling (Amsalem et al., 2014).

The investigation of IIS in insects is presently undergoing enormous progress (for a review see Vafopoulou, 2014). ILPs/IGFs and their specific insulin receptors (InRs) are structural, genetic and functional homologs of mammalian insulins and their receptors. In insects, IIS is involved in the regulation of all main physiological processes, including metabolic aspects of reproduction and diapause. Hence, IIS interacts with other principal signaling pathways, i.e., (1) Target of Rapamycin (TOR), a nutrient sensing responder, (2) JH, the main regulator of development and reproduction, and (3) Forkhead box protein O (FOXO), a transcription factor involved in stress tolerance, diapause, longevity and growth (Vafopoulou, 2014). In consequence, IIS participates in the regulation of insect polyphenism and caste differentiation of eusocial species (e.g., Koyama et al., 2013; Xu et al., 2015). For instance, nutrient sensing triggers IIS activation which induces elevation of JH titers necessary for queen development in honeybee larvae (Mutti et al., 2011). However, honeybees have developed idiosyncrasies that set them apart of the general pattern in insects. JH and vitellogenin (Vg) are negatively correlated in honeybees (Guidugli et al., 2005a) whereas in all other insects studied to date, the inverse pattern was observed (Wyatt and Davey, 1996).

Insect AKHs are peptidic neurohormones, structurally and functionally characterized in a number of solitary insects (Gäde, 2009). Their binding to specific G-protein coupled receptors (AKHRs) activates the mobilization of lipid, carbohydrate and/or proline stores (Gäde and Auerswald, 2003; Van der Horst, 2003). The role of AKH in social Hymenoptera is not well understood. In honeybees, AKH was in some studies either not detected (e.g., Veenstra et al., 2012) or only at low quantities in one subspecies, but not another (Lorenz et al., 1999; Woodring et al., 2003). Also, honeybees seem to not or hardly respond to AKH (Lorenz et al., 1999; Woodring et al., 2003). Similarly, workers of *B. terrestris* and *B. hortorum* did not react to AKH injections (Lorenz et al., 2001), but all investigated bumblebee species contained AKH (Lorenz et al., 2001), and AKH genes are conserved across Hymenoptera (Veenstra et al., 2012). A recent study suggested that IIS and AKHR are possibly co-regulated via JH signaling by means of a putative JH receptor (ultraspiracle, USP) and vitellogenin in honeybee workers (Wang et al., 2012).

Reproductive diapause regulation is thought to be conserved (Sim and Denlinger, 2013), although some taxa are likely to have discrete mechanisms. In bumblebees, the presence of queen diapause has been proposed to be a prerequisite for the evolution of sociality by co-option of the diapause-regulating gene network for new roles in caste differentiation (Hunt and Amdam, 2005; Hunt et al., 2007). Several studies have investigated the genetics underlying diapause in bumblebees (Kim et al., 2006, 2008; Colgan et al., 2011), including a very recent study communicating a genome-wide transcriptome comparison targeting the fat body of diapausing *Bombus terrestris* queens (Amsalem et al., 2015b). The latter study, concurrent with our own investigations, highlighted the functional association of the genes highly expressed in the queen's fat body with nutrient storage and stress resistance, as well as a different role of JH and Vg in diapausing bumblebee queens when compared to dipteran models. This further prompted our interest in a detailed dissection of a more complex set of endocrine markers related to the major transitions in the life of a bumblebee queen.

In the present study, we used qRT-PCR to characterize the expression levels of the following key genes of three major endocrine regulatory pathways in nine tissues, covering virgin, diapausing, and reproducing queens, as well as workers and males in the buff-tailed bumblebee, *B. terrestris*: (1) JH signaling —*methyl farnesoate epoxidase* (*MFE*), *Krüppel homolog 1* (*Kr-h1*) and *vitellogenin* (*Vg*); (2) Insulin signaling/FOXO pathways—*insulin growth factor 1* (*IGF-1*), *locust insulin-related peptide like* (*LIRP-like*), *insulin-like peptide receptor 1* (*InR-1*), *insulin-like peptide receptor 2* (*InR-2*), and *forkhead box protein O* (*FOXO*); (3) AKH signaling—*hypertrehalosaemic prohormone-like* (*AKH*) and *gonadotropin-releasing hormone II receptor-like* (*AKHR* ortholog gene). Expression profiles were characterized in brains, including *corpora allata* (CA) and *corpora cardiaca* (CC), labial glands, hypopharyngeal glands, gonads, flight muscles, the alimentary canal (crop, ventriculus, rectum), and fat bodies.

## Materials and methods

### Bumblebee rearing and collection

Colonies of *B. terrestris terrestris* (buff-tailed bumblebee) were kept at 28°C and a relative humidity of 60% in the laboratories of the Agricultural Research, Ltd., Troubsko, Czech Republic. We sampled five different phenotypes (ten individuals each): (1) Virgin queens collected 7 days after emergence; (2) Diapausing queens—mated queens kept at 4–7°C and sampled after 3 months, i.e., in the middle of hibernation; (3) Reproducing queens—collected from developing colonies with 10 to 15 workers, before the switch point, i.e., before the queen started to produce eggs that develop into haploid males (Duchateau and Velthuis, 1988); (4) Workers—9 days old workers sampled before the switch point and (5) Males—7 days old males, i.e., sexually mature and at the peak of marking pheromone production (Šobotník et al., 2008). The respective castes and queen life stages originated from nests of the same lineage of *B. terrestris terrestris*. Immediately after collection, individuals were frozen in crushed dry ice and stored at −80°C until dissections took place. This study complies with the current laws in the Czech Republic; no ethical approval was required for this study on laboratory-bred insects.

### Tissue samples preparation

Individuals were dissected under a stereomicroscope on sterilized glass Petri dishes placed on crushed ice and in sterile, ice-cold RNAase-free Ringer solution. In order to map the organ-specific expression profile of investigated genes, we dissected nine different tissues from each individual: (1) brain—brain including both paired glands *corpora cardiaca* (CC) and *corpora allata* (CA); (2) hypopharyngeal and (3) labial glands—paired tissues from the head capsule; (4) flight muscles; (5) crop; (6) ventriculus; (7) rectum; (8) gonads (ovaries or testes); and (9) abdominal fat body—peripheral fat body attached to epidermis (see Supplementary Figure 1). Two samples of each tissue were pooled as one replicate, i.e., altogether five biological replicates per tissue of each *B. terrestris* phenotype were analyzed. Whereas the ovaries in virgins and diapausing queens were shrunk and transparent, ovary development of workers corresponded to stages I–II of oogenesis process (Duchateau and Velthuis, 1989). Immediately after dissection, all tissues were transferred to microcentrifuge tubes with 200 μL of TRI Reagent® (Sigma-Aldrich) on crushed ice and then stored at −80°C until RNA isolation.

### RNA isolation and cDNA synthesis

We extracted total RNA using TRI Reagent® (Sigma-Aldrich) following the manufacturer's protocol. RNA isolates were treated with RQ1 RNase-Free DNase (Promega) to remove traces of contaminant DNA. The cDNA template was prepared using the SuperScript® III First-Strand Synthesis System for RT-PCR (Invitrogen by Life Technologies) on 400 ng of the corresponding total RNA with random hexamers. Primer pairs efficiency was tested using cDNA mixtures of all biological samples serially diluted into five concentrations and subsequently constructed standard curves (for selected primer pairs sequences and their respective efficiency values see Supplementary Table S1). Prior to qRT-PCR, cDNAs of all samples and the calibrator cDNA (i.e., the mixture of aliquots from all samples) were diluted ten-fold.

### qRT-PCR analysis

We performed qRT-PCR using a LightCycler 480 qRT-PCR System (Roche) with SYBR green fluorescent labels and 200 nM of each primer. The PCR program was as follows: Initial denaturation for 15 min at 95°C, followed by 40 cycles of 30 s at 95°C, 30 s at 55°C and 30 s at 72°C. The final extension was at 72°C for 2 min. A final melt-curve step was included post-PCR (ramping from 55°C to 95°C at 0.1°C steps every 5 s) to confirm the absence of any non-specific amplification. All biological samples were examined in two technical replicates. In order to allow comparison of all tested samples, calibrator cDNA was applied to a master-mix of specific genes on each plate. To obtain relative expression values for each target gene, generated Cp values were calibrated and normalized using the geometric mean of phospholipase A2 (*PLA2*) and elongation factor eEF-1 α (*EF1*) (Pfaffl, 2001). We selected housekeeping genes based on previous studies (Hornáková et al., 2010; Verlinden et al., 2013) and on the evaluation of *PLA2, EF1* and *arginine kinase* using qRT-PCR across all biological samples using NormFinder (Andersen et al., 2004), in which *PLA2* and *EF1* were found to be the best combination for our experimental setup.

### Sequence analyses

Insulin-like peptide sequences were analyzed using programs available at the Biology workbench server (http://workbench.sdsc.edu). Pairwise protein sequence alignment was performed using the EMBOSS Stretcher tool (http://www.ebi.ac.uk/Tools/psa/emboss_stretcher/).

### Data processing and statistics

Results were plotted using the Prism graphic program (GraphPad Software, version 5.0, San Diego, CA, USA). The bars represent the mean of measurements ± SEM (*n* = 3–5). In order to process data with normal distribution, the results were log-transformed and then evaluated by one-way ANOVA with Tukey multiple comparison *post-hoc* test (*p* < 0.05). We corrected for multiple testing using the Benjamini-Hochberg-method implemented in R. We generated heatmaps in R 3.2.3 using R package “gplots” (R. Development Core Team, 2015; Warnes et al., 2015). The matrix of log-transformed mean values of each gene's expression per specific phenotype and tissue was used for heatmap constructions.

## Results and discussion

### JH signaling

**Figure 1:**
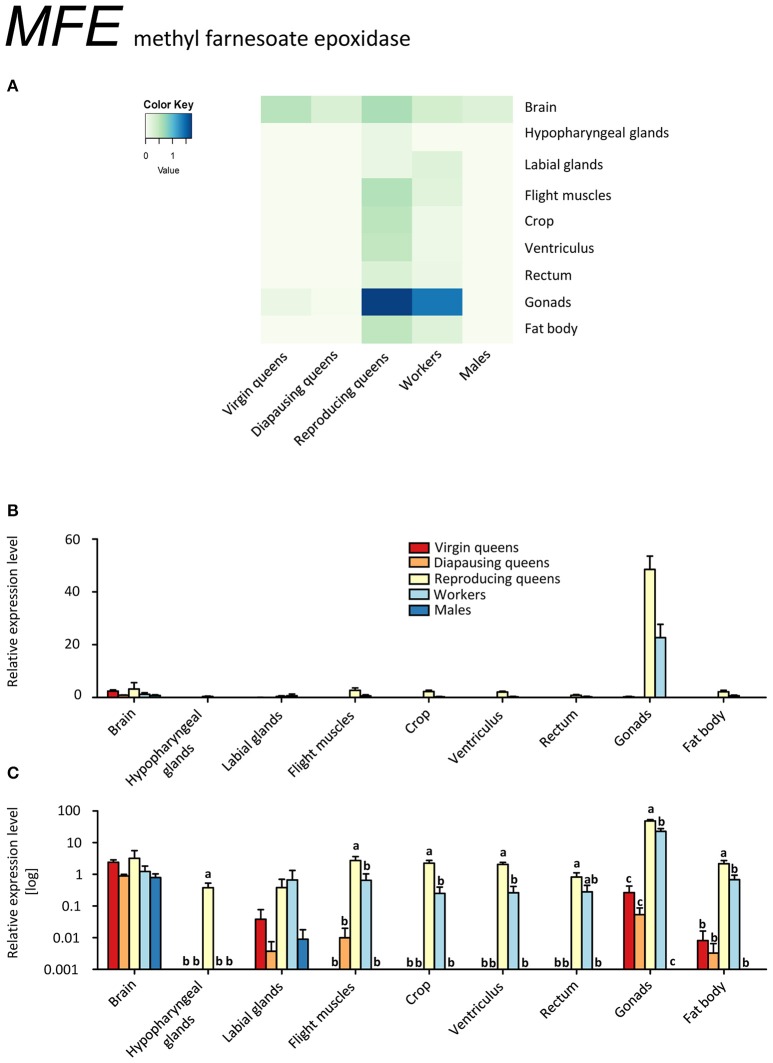
**qRT-PCR analysis of tissue-specific relative expression levels of *methyl farnesoate epoxidase* (*MFE*) across castes of *B. terrestris***. MFE is crucial in synthetizing juvenile hormone (JH). It is heavily upregulated in gonads of workers and reproducing queen. It is also found in brains of all samples (see main text). Data represent mean ± SEM (*n* = 3–5) of normalized and rescaled expression levels. **(A)** Heatmap: Log-scale. Bar graphs: Relative transcript levels in linear scale **(B)** and log-transformed scale **(C)**. Significantly different expression levels are indicated by different letters (*p* < 0.05).

The highest transcript levels of *methyl farnesoate epoxidase* (*MFE*) were found in ovaries of workers and reproducing queens (Figures 1A–C, Supplementary Table S2). This is striking, since JH synthesis usually takes place in the *corpora allata* (CA) (e.g., Jindra et al., 2013) and occurs in the gonads in only a few species (reviewed in De Loof et al., 2013). Yet, since the alternative JH production sites are only poorly investigated so far, it is possible that the production of at least some JH in the ovaries may be more widespread than previously considered (De Loof et al., 2013). Even though a high JH synthesis is not automatically synonymous with elevated JH titers (Bloch et al., 2000), our results support the previously proposed conservation of the gonadotropic role of JH in bumblebees (Bloch et al., 2000; Shpigler et al., 2014, 2016; Amsalem et al., 2015b).

The ability of reproducing queens and workers to produce JH in ovaries does not imply the presence of the whole JH biosynthesis pathway in this tissue. Strictly speaking, our results only indicate a high conversion rate of methyl farnesoate to JH in ovaries. A similar process, namely the conversion of JH acid to JH by juvenile hormone acid methyltransferase (JHAMT), was described in imaginal discs of post-wandering larvae of the tobacco hornworm, *Manduca sexta* (Sparagana et al., 1985). Potentially, CA release not only JH but also methyl farnesoate into the hemolymph that is then converted into JH by MFE in the ovaries. Therefore, our finding of high *MFE* expression in ovaries of bumblebee females may explain the reported discrepancy between low JH biosynthesis rates in CA but high JH titers and developed ovaries (Bloch et al., 2000). Allatectomized workers had greatly reduced JH titers in the hemolymph compared to controls (Shpigler et al., 2014), which could be explained by a lack of substrate for MFE under this scenario. The role of JH in the ovaries might be also to act locally in order to enhance vitellogenin uptake, as has been described for *Dysdercus koenigii* (Venugopal and Kumar, 2000) and *Solenopsis invicta* (Vargo and Laurel, 1994), without being secreted to the hemolymph.

Eight out of ten workers had developed ovaries in various stages; likely they were preparing to compete with the queen for male parentage (Duchateau and Velthuis, 1988). Their expression profile for *MFE* closely resembled that of fertile queens, consistent with the report by Harrison et al. (2015) who suggested that reproductive workers generally become more queen-like. This pattern, however, was not observed for the other genes under investigation. In general, our findings contrast with the situation in the honeybee (Bomtorin et al., 2014; Niu et al., 2014), in which almost no expression of *MFE* and relatively low expression of the direct upstream enzyme *JHAMT* were detected in ovaries (Bellés et al., 2005). This conspicuous difference is interpreted as an uncoupling of JH from the gonadotropic functions in the highly eusocial honeybees (Hartfelder and Emlen, 2012).

Gonads of virgin and diapausing queens had only low transcript levels, and males showed no expression outside the brain tissue (Figures 1A–C, Supplementary Table S2). *MFE* expression was relatively low in the brains of all samples. In hypopharyngeal glands, expression was absent except for low values in reproducing queens. Workers and reproducing queens showed low *MFE* expression values also in the remaining tissues, whereas virgin and diapausing queens showed spurious or no expression.

**Figure 2:**
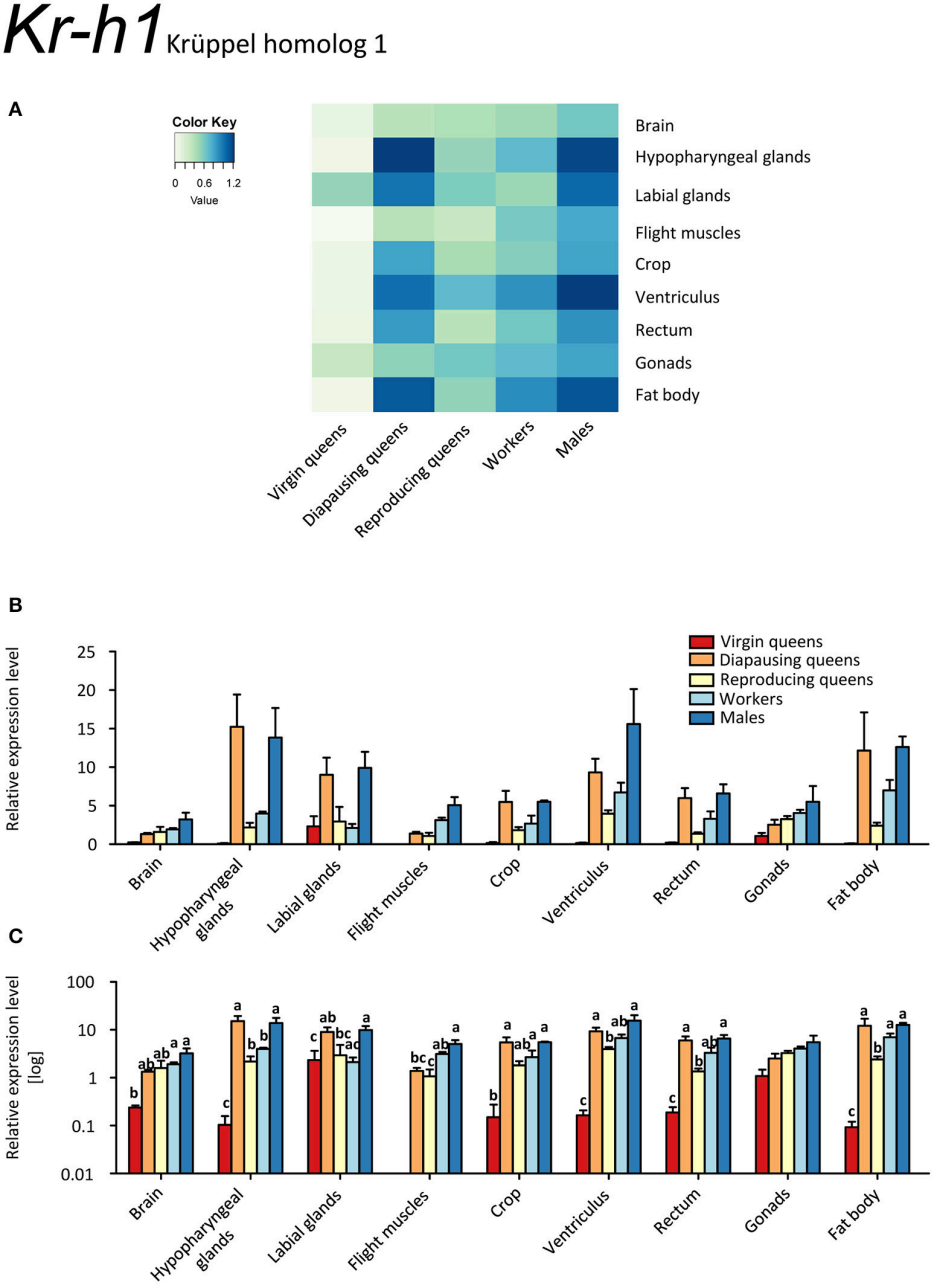
**qRT-PCR analysis of tissue-specific relative expression levels of *Krüppel homolog 1* (*Kr-h1*) across castes of *B. terrestris***. Kr-h1 is a transcription factor downstream of juvenile hormone receptors. It is downregulated in virgin queens, and upregulated in males and diapausing queens (see main text). Data represent mean ± SEM (*n* = 3–5) of normalized and rescaled expression levels. **(A)** Heatmap: Log-scale. Bar graphs: Relative transcript levels in linear scale **(B)** and log-transformed scale **(C)**. Significantly different expression levels are indicated by different letters (*p* < 0.05).

Our results indicate only low expression of *Kr-h1* in virgin queens, except for slightly elevated values in the labial glands, and even lower expression in ovaries (Figures 2A–C, Supplementary Table S2). This is in stark contrast with the other females showing considerably higher expression levels, and to males who had the highest expression values in all tissues but labial glands. Reproducing queens were somewhat intermediary between the very low levels in virgins and the rather high levels in diapausing queens. Across all samples, particularly high expression occurred in labial and hypopharyngeal glands, ventriculus, and fat body.

**Figure 3:**
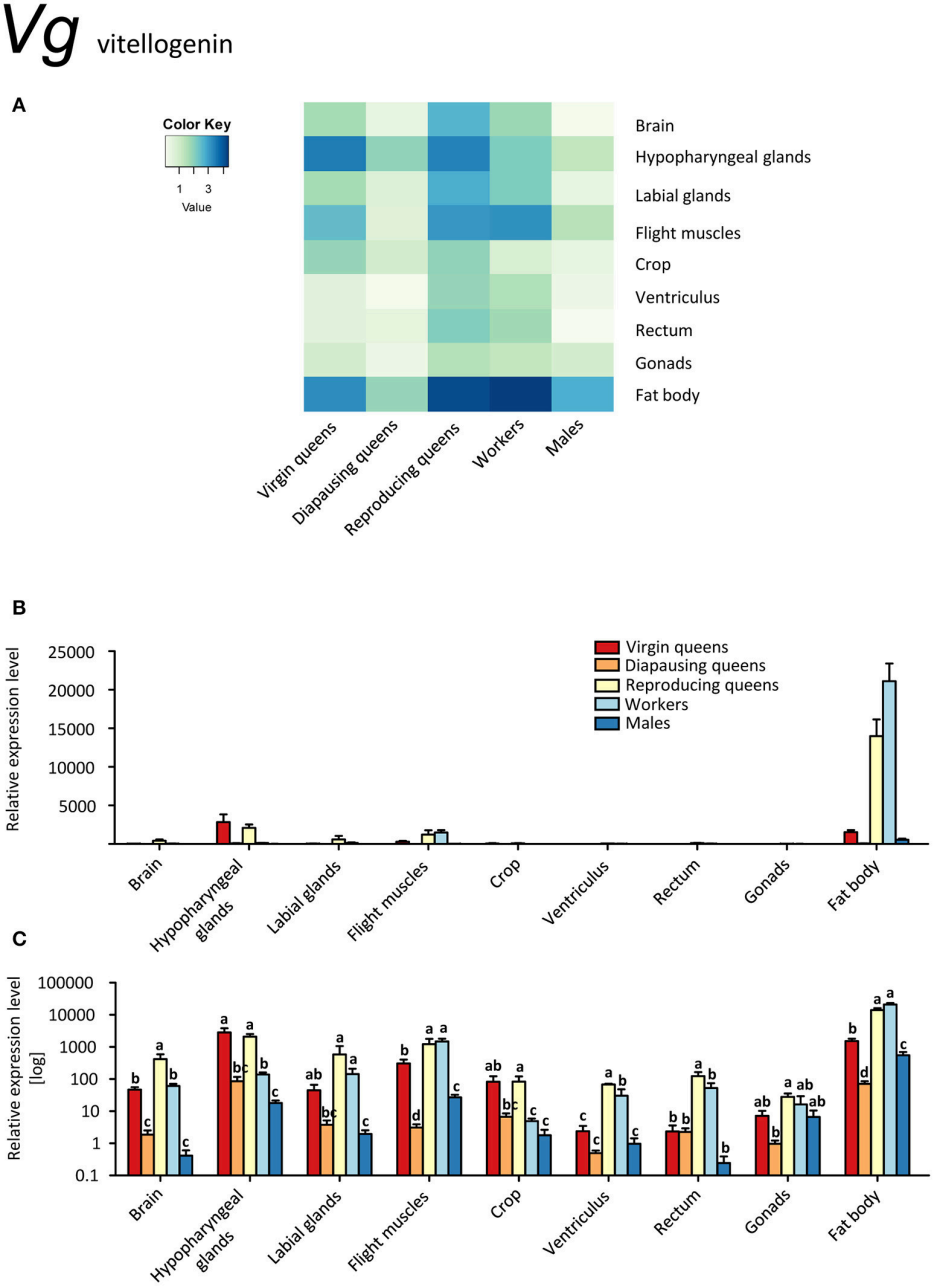
**qRT-PCR analysis of tissue-specific relative expression levels of *vitellogenin* (*Vg*) across castes of *B. terrestris***. Vg is a yolk protein with multiple functions in immunity and behavioral regulation. It is highly upregulated in fat body of all castes, and generally lowest in males and diapausing queens (see main text). Data represent mean ± SEM (*n* = 3–5) of normalized and rescaled expression levels. **(A)** Heatmap: Log-scale. Bar graphs: Relative transcript levels in linear scale **(B)** and log-transformed scale **(C)**. Significantly different expression levels are indicated by different letters (*p* < 0.05).

Among the studied queen life stages, the highest *Kr-h1* expression was recorded in most tissues of diapausing queens, except for flight muscles and ovaries (Figures 2A–C, Supplementary Table S2). Conversely, virgins and reproducing females had low expression of *Kr-h1*. This expression pattern contrasts sharply with that of the two other markers of JH signaling, *MFE* and *Vg* (Figures 1A–C, 3A–C, respectively). This finding is also contradictory to the only available comparative data on females of the red firebug, *Pyrrhocoris apterus* (Bajgar et al., 2013), where *Kr-h1* levels were low in diapausing females and increased immediately in response to external application of JH mimics. On the other hand, a recent work revealed that mating can trigger *Kr-h1* upregulation in the gut of *Drosophila melanogaster* females together with gut tissue remodeling for increased lipid metabolism (Reiff et al., 2015). Thus, one may speculate whether Kr-h1 brings about tissue reconstruction in queens after mating during the course of diapause. Nevertheless, at the present state of knowledge, we are still far from a satisfactory explanation for why *Kr-h1*'s expression pattern is incongruent with other JH signaling markers.

Vitellogenins, large phosphoglycolipoproteins, have been attributed a plethora of functions. They are best known for their role as yolk precursors in oviparous animals (Valle, 1993). Additionally, they also act as carriers for hormones, vitamins, metals, and others (reviewed in Sappington and Raikhel, 1998). Vg shows antimicrobial properties and is involved in immunity (Amdam et al., 2004; Sun and Zhang, 2015), it has anti-oxidative (Havukainen et al., 2013; Sun and Zhang, 2015), and potentially anti-inflammatory properties (Havukainen et al., 2013; Salmela et al., 2016). Further, expression of *Vg* genes correlates with castes and behavior in some ants (Wurm et al., 2011; Corona et al., 2013) and might be involved in caste determination in ants (Libbrecht et al., 2013) and honeybees (Barchuk et al., 2002). Thus, Vg clearly has many more functions than being a yolk precursor only. Moreover, as in other hymenopterans, three *Vg*-like genes with as yet unknown function are present in several bumblebee species, including *B. terrestris* (Harrison et al., 2015), suggesting that these gene duplications allowed new functions to evolve (Morandin et al., 2014). Among them the reproducing female specific gene marked “*Vg1*” (Morandin et al., 2014; Harrison et al., 2015) is the gene variant studied in this work.

*Vg* was expressed in all studied tissues (Figures 3A–C). Reproducing queens showed the highest, whereas diapausing queens and males the lowest expression levels. As expected, the strongest expression occurred in the fat bodies, particularly in reproducing queens and workers (up to 38 times more transcripts than in virgins and males; Figures 3B,C).

Our data on *MFE* and *Vg* expressions (Figures 1A–C, 3A–C) are in line with the gonadotropic function of JH signaling that leads to increased vitellogenin synthesis in reproducing females (Amsalem et al., 2014; Shpigler et al., 2014). Their low expression levels during diapause are characteristic for a reproductive arrest in hibernating insects (e.g., Goodman and Cusson, 2012). Our findings contrast with the data of Amsalem et al. (2015b) who found no differences in *Vg* expression in fat bodies of mated, diapausing queens, and post diapause foundresses. Potentially, these differences stem from the time of sampling, i.e., before (Amsalem et al., 2015b) and after the emergence of workers (the present study). Amsalem et al. (2015b) suggested this discrepancy with most insects might be a sign of derived functions of vitellogenin in social insects. This view is partially supported by our observations in other tissues.

Besides fat body tissues, also brains, both glands and flight muscles manifested a considerable upregulation. While in the fat body Vg likely serves the canonical storage and yolk protein role, its ubiquitous expression in virtually all tissues, including brain, suggests additional functions. Interestingly, higher *Vg* expression was found in brains and hypopharyngeal glands of reproducing queens compared to workers with vitellogenic ovaries (i.e., 7 and 15 times in brains and hypopharyngeal glands, respectively; Figures 3A–C, Supplementary Table S2). These are the only tissues with *Vg* upregulated in comparison with workers. Since *Vg* expression in the brains of some insects is linked to caste- or reproduction-related behavior and physiology (Corona et al., 2007; Roy-Zokan et al., 2015), it appears likely that this finding could be linked to a queen-specific role of Vg.

In most insects, JH and Vg are positively correlated, but there are also cases where Vg became uncoupled or even interacts negatively with JH (Amsalem et al., 2014 and references therein). Here, we confirm that Vg correlates with egg-laying in bumblebee queens (Amsalem et al., 2014; Harrison et al., 2015). Our results are largely in agreement with data on whole body extracts (Harrison et al., 2015) and head extracts (Amsalem et al., 2014), reporting higher *Vg* expression in the heads of reproducing queens than in virgins and sterile or fertile workers. The observed differences between tissues in our study demonstrate the value of detailed investigations. For instance, we found *Vg* transcripts in varying abundances in the brain, labial glands, and the highest expression in hypopharyngeal glands, all situated within the head capsule. Vg protein has been detected in honeybee nurse head fat body and likely in glia cells (Seehuus et al., 2007; Münch et al., 2015). Reports of Vg transcripts in honeybee brain might be due to contamination with head adipocytes (Münch et al., 2015), but could as well signal genuine, probably also age-dependent, though rather low transcription levels. The function of Vg in glia cells or the brain in general remains unknown; it has been speculated that they might suppress inflammatory processes (Münch et al., 2015).

The fat body is regarded as the main location of Vg synthesis (e.g., Arrese and Soulages, 2010). Also in the present study, transcript levels were ca. 7 times higher in the fat body than in tissues with the next highest expression (hypopharyngeal glands, labial glands, flight muscles).

We show that *Vg* transcripts are also found in male and female gonads. Vitellogenesis in ovaries is not unusual in other insects (reviewed in Valle, 1993), and honeybee queens do express *Vg* in their ovaries as well (Guidugli et al., 2005b). Guidugli et al. (2005b) could not detect *Vg* expression in worker ovaries; however, it would seem they investigated only sterile workers, and we expect the fertile workers to express *Vg* in ovaries. Seehuus et al. (2007) confirmed the presence of Vg proteins in worker ovaries, but did not study their origin.

In insects, Vg is not common in males, but not exceptionally rare (reviewed in Valle, 1993); in social insects it occurs for instance in males of termites (Weil et al., 2009), stingless bees (Dallacqua et al., 2007), honeybees (Colonello-Frattini et al., 2010), and two species of bumblebees (Li et al., 2010; Harrison et al., 2015).

Unlike in honeybees, where *Vg* mRNA levels in abdomens, fat body and male accessory glands were only moderate in 1-day-old drones and then declined (Piulachs et al., 2003; Corona et al., 2007; Colonello-Frattini et al., 2010), we found very high transcript levels of *Vg* in fat bodies of mature males. Additionally, *Vg* was expressed in hypopharyngeal glands, flight muscles, and gonads. This supports once again the expected pleiotropic function of Vg. Colonello-Frattini et al. (2010) interpret the finding of Vg in male gonads as a “cross-sexual transfer” from traits evolved in female castes. Piulachs et al. (2003) argue that it is not a partial development of female traits, because it is also found in diplo-diploid species, and suggest that in Hymenoptera at least, it is related to sociality. Currently, there are not enough studies on non-social Hymenoptera to verify this hypothesis.

Cephalic labial glands are well known as the synthesis site of sex (marking) pheromones in bumblebee males (Žáček et al., 2013). Their role in females, where they are drastically reduced, remains elusive.

Unlike in honeybees, hypopharyngeal glands are developed in all bumblebee castes and in both sexes. Similar to honeybees, these glands produce a BtRJPL (*B. terrestris* royal jelly protein-like) protein, an ortholog of honeybee major royal jelly proteins (Kupke et al., 2012). Although these proteins are orthologs, the exact physiological function in bumblebees remains poorly understood (Albert et al., 2014). Likely, the ancestral role of major royal jelly proteins was pre-digestive food modification, comparable to saliva (Kupke et al., 2012), and the nutritive function in honeybees is derived. Similarly, the hypopharyngeal glands secretion contains digestive enzymes such as amylase and invertase (Pereboom, 2000). Nevertheless, the bumblebee hypopharyngeal glands might also serve as storage for Vg, analogous to the fat body, as was suggested for honeybees by Amdam and Omholt (2002).

Head fat body tissue was clearly visible in our dissections and firmly attached to the chitinous elements in the head. While we cannot rule out that some adipocytes might have been sticking to brains or labial glands during the dissections (compare Münch et al., 2015), this would not explain the high expression levels in hypopharyngeal glands. In general, the ubiquitous occurrence of Vg in a variety of different tissues suggests that it might serve as regulator of immune functions, including anti-inflammatory and anti-oxidative defense, as reported for honeybees (Amdam et al., 2004; Havukainen et al., 2013).

### Insulin signaling

A search for putative homologs of known insect genes of IIS in the *B. terrestris* genome using BLAST (Madden, 2003) unveiled two pairs of insulin peptides and receptors (see Supplementary Table S1). These were *insulin-like growth factor 1* (*IGF-1*) and *locust insulin-related peptide-like* (*LIRP*) as the predicted ligands, and *insulin-like peptide receptors InR-1* and *InR-2*. The sequence analysis of the two *B. terrestris* insulin peptides revealed high identity with the IGF-1 and LIRP from *Apis florea*. Similarily, their sequences matched with the respective homologs of human IGF-1 and *Locusta migratoria* LIRP (see Supplementary Figure 2). Therefore, we use the terms “IGF-1” and “LIRP”, respectively, for these two peptides.

**Figure 4:**
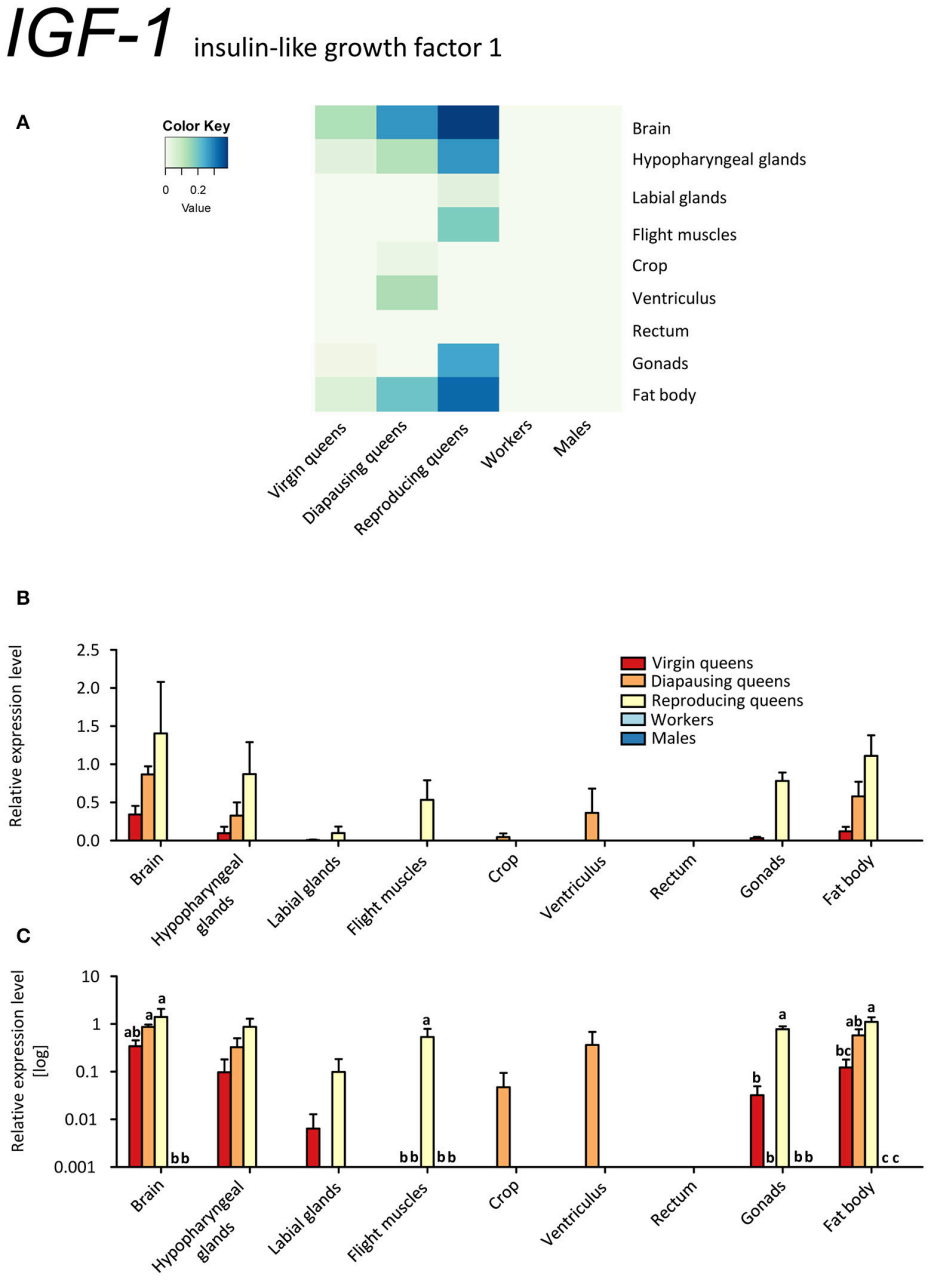
**qRT-PCR analysis of tissue-specific relative expression levels of *insulin-like growth factor 1* (*IGF-1*) across castes of *B. terrestris***. IGF-1 is part of the IIS signaling involved in reproduction and diapause. It is queen-specific (i.e., absent in workers and males) and lowest in virgin queens (see main text). Data represent mean ± SEM (*n* = 3–5) of normalized and rescaled expression levels. **(A)** Heatmap: Log-scale. Bar graphs: Relative transcript levels in **(B)** linear scale; and **(C)** log-transformed scale. Significantly different expression levels are indicated by different letters (*p* < 0.05).

*IGF-1* was expressed exclusively in queens (Figures 4A–C). In virgins, only spurious expression was detected in brain, hypopharyngeal glands, fat body, and ovaries. Higher expression values were observed in diapausing queens, where the ventriculus also contained *IGF-1* transcripts. The highest expression occurred in brain, hypopharyngeal glands and fat body tissues of reproducing queens (~2 to 3 times higher than in diapausing queens and 4 to 10 times higher than in virgins; Figures 4A–C, Supplementary Table S2). Here, *IGF-1* was present in all investigated tissues except for the digestive tract.

**Figure 5:**
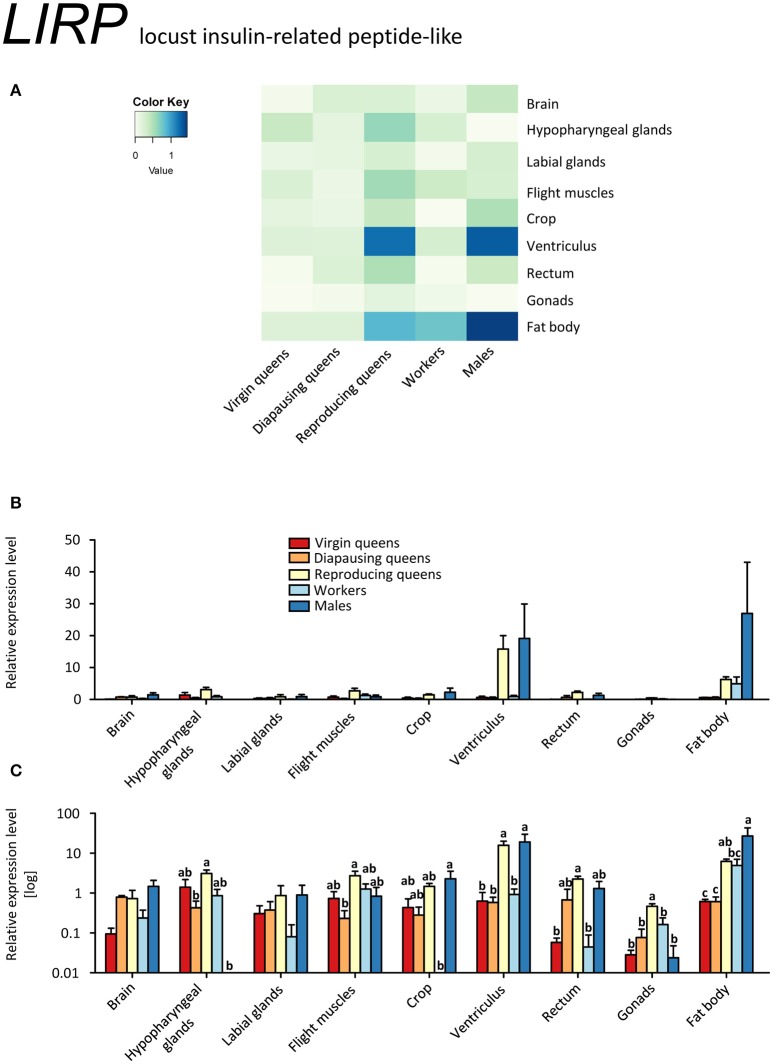
**qRT-PCR analysis of tissue-specific relative expression levels of *locust insulin-related peptide-like 1* (*LIRP*) across castes of *B. terrestris***. LIRP is part of the insulin signaling involved in reproduction and diapause. It is most abundant in fat body and ventriculus of males and reproducing queens (see main text). Data represent mean ± SEM (*n* = 3–5) of normalized and rescaled expression levels. **(A)** Heatmap: Log-scale. Bar graphs: Relative transcript levels in linear scale **(B)** and log-transformed scale **(C)**. Significantly different expression levels are indicated by different letters (*p* < 0.05).

In contrast to *IGF-1, LIRP* was expressed in virtually all samples, except for males' hypopharyngeal glands and workers' crop (Figures 5A–C, Supplementary Table S2). This is consistent with the findings in desert locusts, *Schistocerca gregaria* (Badisco et al., 2008). The highest expression levels occurred in the ventriculus and fat body of reproducing queens and males. Reproducing queens had often higher expressions than virgins or diapausing queens (Figures 5A–C, Supplementary Table S2).

Since transcripts of both *B. terrestris* insulin-related peptides are the most abundant in reproducing queens, the IIS in bumblebees is apparently correlated with upregulated JH signaling and high nutritional and reproductive status, as is also reported for solitary insects (Badisco et al., 2013). This is supported by increased *IGF-1* expression in reproducing queens, which correlates with the pattern of *Vg* in the same tissues, especially in brain and hypopharyngeal glands, flight muscles and fat body (compare Figures 3A–C, 4A–C). However, *IGF-1* transcripts are absent in workers with developing ovaries showing an upregulated JH signaling (*Vg* and *MFE* expression). We hypothesize that suppressed *IGF-1* expression in workers is linked to the developmental arrest in vitellogenic ovaries in queen-right colonies.

**Figure 6:**
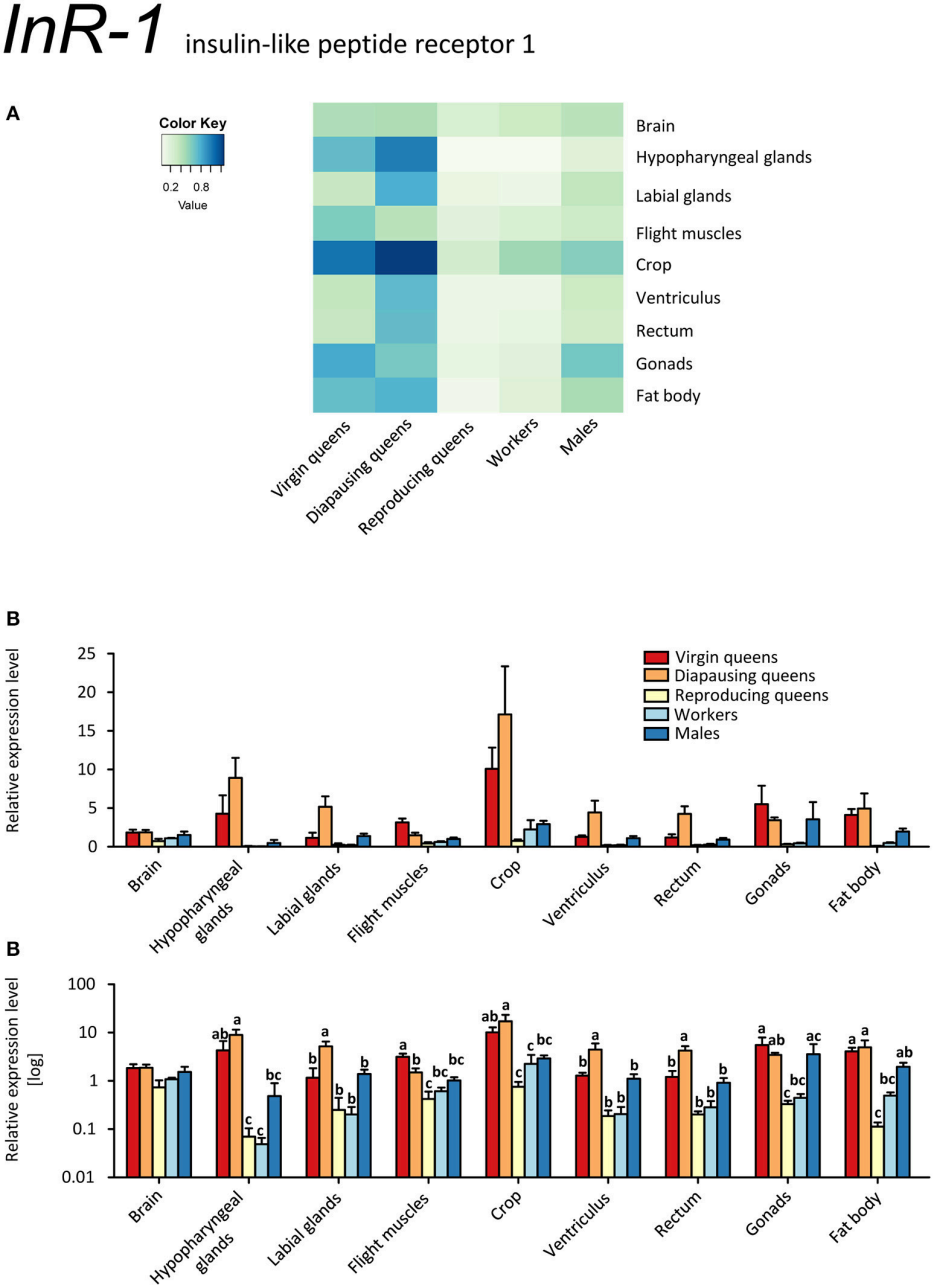
**qRT-PCR analysis of tissue-specific relative expression levels of *insulin-like peptide receptor 1* (*InR-1*) across castes of *B. terrestris***. InR-1 is a receptor involved in insulin signaling. It is upregulated in virgin and diapausing queens, especially in crop and hypopharyngeal glands (see main text). Data represent mean ± SEM (*n* = 3–5) of normalized and rescaled expression levels. **(A)** Heatmap: Log-scale. Bar graphs: Relative transcript levels in linear scale **(B)** and log-transformed scale **(C)**. Significantly different expression levels are indicated by different letters (*p* < 0.05).

*InR-1* transcripts were detected in all samples. By far the highest expression occurred in tissues of virgin and diapausing queens (Figures 6A–C). By contrast, reproducing queens showed the lowest expression values (for all tissues except cephalic glands; see Figures 6A–C). The *InR-1* expression pattern in queens is inverse to the expression of its putative ligands IGF-1 and LIRP (compare Figures 4A–C, 5A–C, and 6A–C). Unexpectedly high expression occurs in the crop in all stages but the reproducing queens. Males' testes also showed *InR-1* transcript levels as elevated as in males' crop.

**Figure 7:**
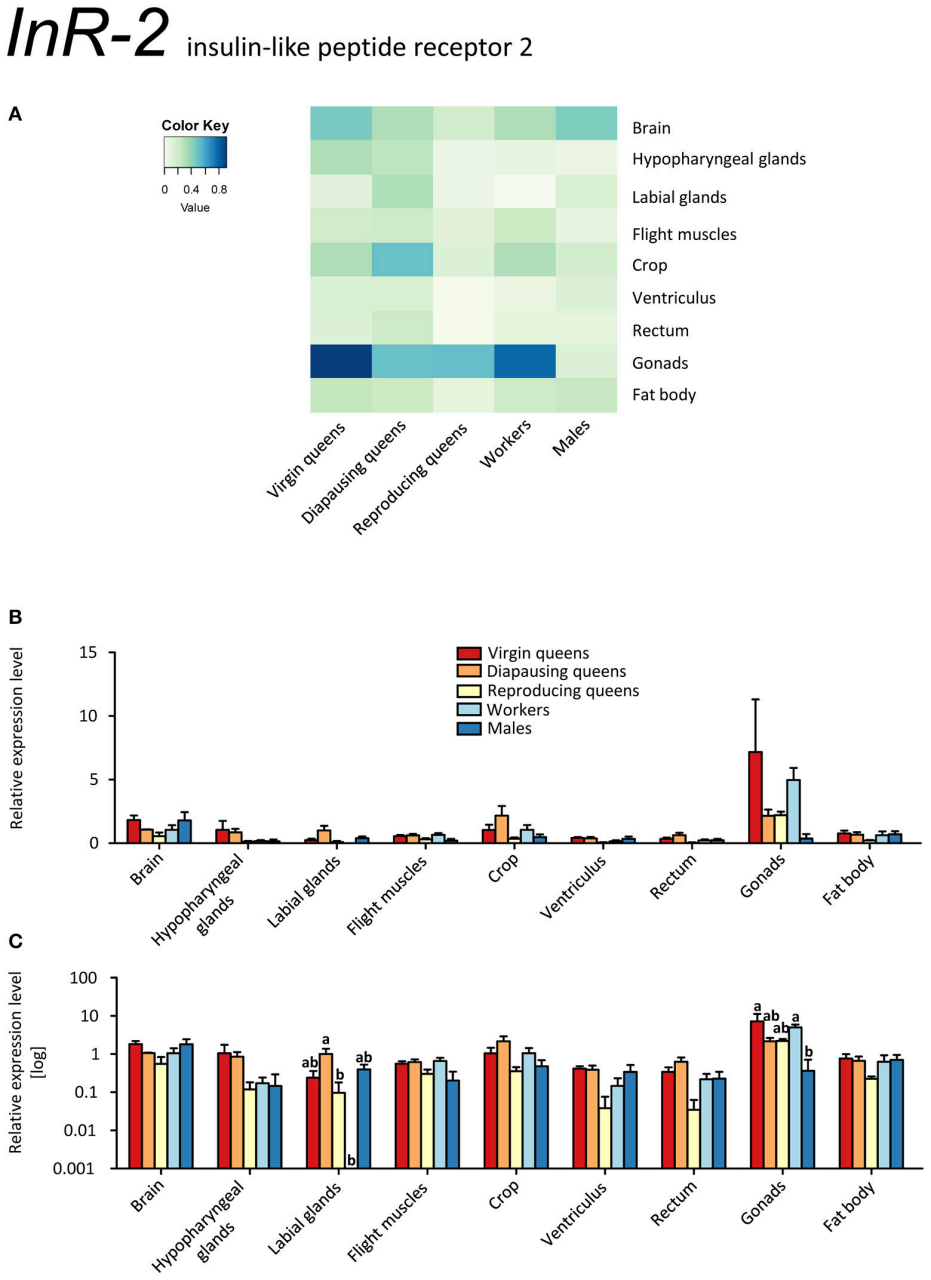
**qRT-PCR analysis of tissue-specific relative expression levels of *insulin-like peptide receptor 2* (*InR-2*) across castes of *B. terrestris***. InR-2 is a receptor involved in insulin signaling. It is upregulated in gonads (see main text). Data represent mean ± SEM (*n* = 3–5) of normalized and rescaled expression levels. **(A)** Heatmap: Log-scale. Bar graphs: Relative transcript levels in linear scale **(B)** and log-transformed scale **(C)**. Significantly different expression levels are indicated by different letters (*p* < 0.05).

*InR-2* was expressed ubiquitously, but at low levels, in all stages, except for workers' labial glands (no transcript detected) (Figures 7A–C, Supplementary Table S2). The highest expression occurred in the ovaries of all females. The high expression levels in the gonads might point at a role in reproduction, because IIS controls synthesis of JHs and ecdysteroids, i.e., the main regulators of insect reproductive physiology (reviewed in Antonova et al., 2012; Badisco et al., 2013). Yet, it is clear that *InR-2* plays not a single role, given the widespread expression across almost all tissues. If *InR-2* is indeed involved in reproduction, the elevated expression in workers' gonads would suggest once again that some workers were prepared to reproduce during the competition phase (Duchateau and Velthuis, 1988).

Expressions of *InRs* were studied in two other hymenopterans, *A. mellifera* and the fire ant *Solenopsis invicta* (Ament et al., 2008; de Azevedo and Hartfelder, 2008; Lu and Pietrantonio, 2011). Similar to the *InR-1* findings shown in the present study, *InRs*' transcripts were more abundant in virgin queens of *S. invicta* compared to workers (Lu and Pietrantonio, 2011). In *A. mellifera*, IIS genes were upregulated in foragers vs. nurses, whereas the expression of *AmAKH* and *AmAKHR* did not differ significantly (Ament et al., 2008; de Azevedo and Hartfelder, 2008). However, these reports focused on honeybee phenotypes with regard to age polyethism and thus are difficult to compare with our worker sampling.

Most insects express one insulin receptor which is likely to bind the insulin-like peptides and insulin growth factors (Okamoto et al., 2009). For instance, the insulin receptor identified in *Drosophila* (dInR) binds the insulin-like peptides dILP1-5 as well as the insulin growth factor-like peptide dILP6 (Brogiolo et al., 2001; Okamoto et al., 2009). Thus, we can anticipate that also InR-1 and InR-2 are activated by either IGF-1 or LIRP.

**Figure 8:**
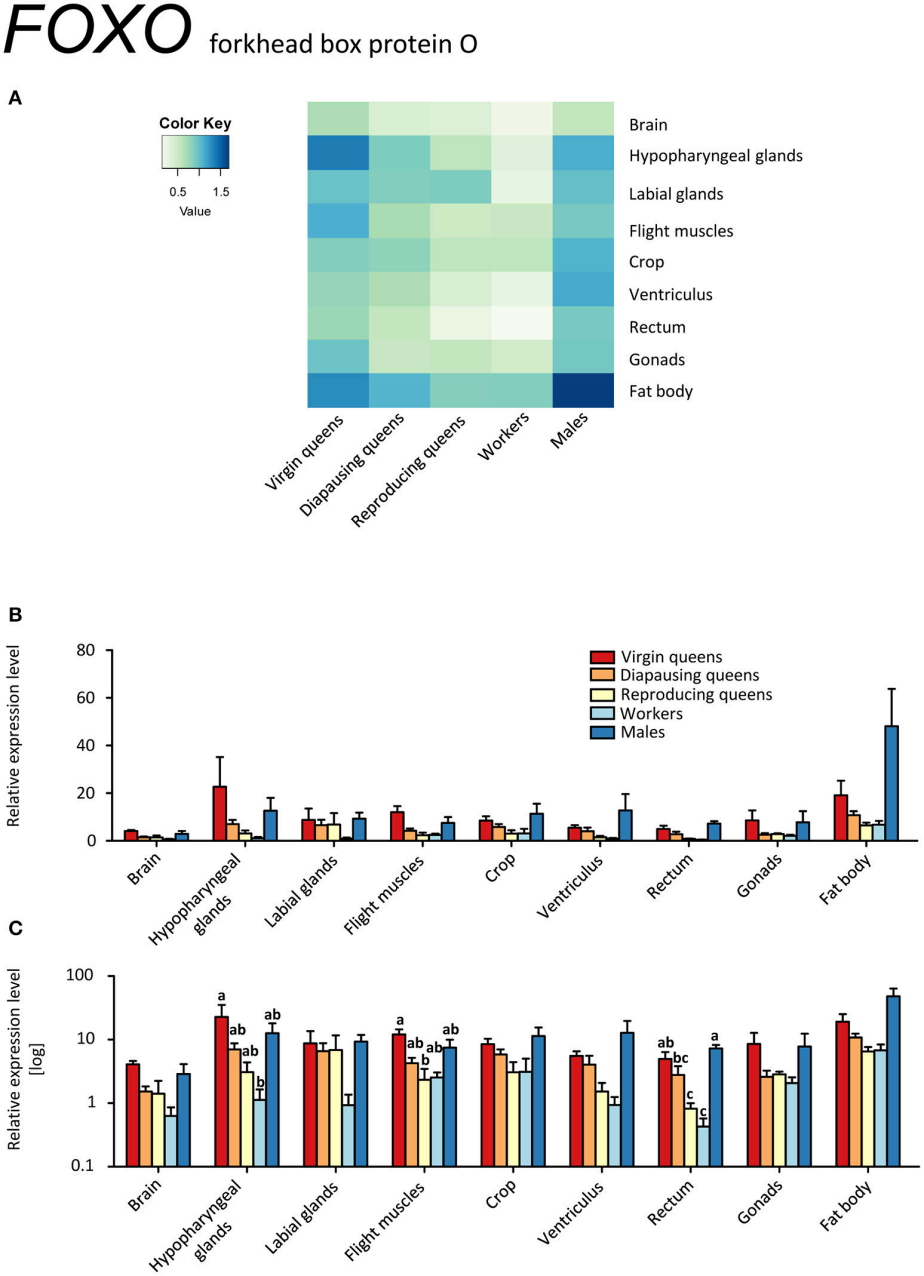
**qRT-PCR analysis of tissue-specific relative expression levels of *forkhead box protein O* (*FOXO*) across castes of *B. terrestris***. FOXO is a transcription factor involved in stress tolerance, diapause, longevity, and growth. *FOXO* transcription is lower in workers and reproducing queens than in other castes (see main text). Data represent mean ± SEM (*n* = 3–5) of normalized and rescaled expression levels. **(A)** Heatmap: Log-scale. Bar graphs: Relative transcript levels in linear scale **(B)** and log-transformed scale **(C)**. Significantly different expression levels are indicated by different letters (*p* < 0.05).

Interestingly, we found opposite expression levels of insulin-like peptides and their respective receptors. Whereas the genes coding for both ligands (*IGF-1, LIRP*) had high expression, *InR-1* showed the lowest expression (reproducing queens). Conversely, low *IGF-1* and *LIRP* transcript levels occurred when *InR-1* was upregulated in the same tissue (virgins, compare Figures 4A–C and 6A–C). It was previously shown that “both in *Drosophila* and mammals, insulin receptor (InR) represses its own synthesis by a feedback mechanism directed by the transcription factor dFOXO/FOXO1” (Puig and Tjian, 2005). Specifically, in *Drosophila*, dFOXO directly activates *dInR* transcription, and its action can be modulated by nutritional status. This is consistent with our records in virgins as *FOXO* and *InR-1* transcriptions were upregulated simultaneously, whereas *IGF-1* and *LIRP* showed very low expression across investigated tissues (Figures 4A–C, 5A–C, 6A–C, and 8A–C).

It is tempting to propose that the extremely high expression of *InR-1* in all tissues of virgins and diapausing queens compared to all other samples is related to their high food (energy) intake prior to diapause by virgins and its consequent preservation by diapausing queens (Badisco et al., 2013). By contrast, reproducing queens have relatively low level of *InR-1* transcripts, indicating that they are not storing nutrients but rather spending them.

The FOXO transcription factor is situated downstream of IIS and JH signaling, and is considered to be the main regulator of insect diapause which includes, among others, cell cycle arrest and fat accumulation in early diapause stage (reviewed in Sim and Denlinger, 2013). Besides diapause, FOXO controls stress tolerance, longevity, and growth in insects (reviewed in Vafopoulou, 2014). In our model, *FOXO* was expressed in all investigated tissues (Figures 8A–C). Elevated expression occurred across tissues of males and virgins, especially in fat body and hypopharyngeal glands. We hypothesize that higher *FOXO* expression in virgins' tissues correlates with its regulatory role in energy reserve accumulation during the prehibernation stage, as is also suggested by high *InR-1* levels.

### AKH/AKHR signaling

**Figure 9:**
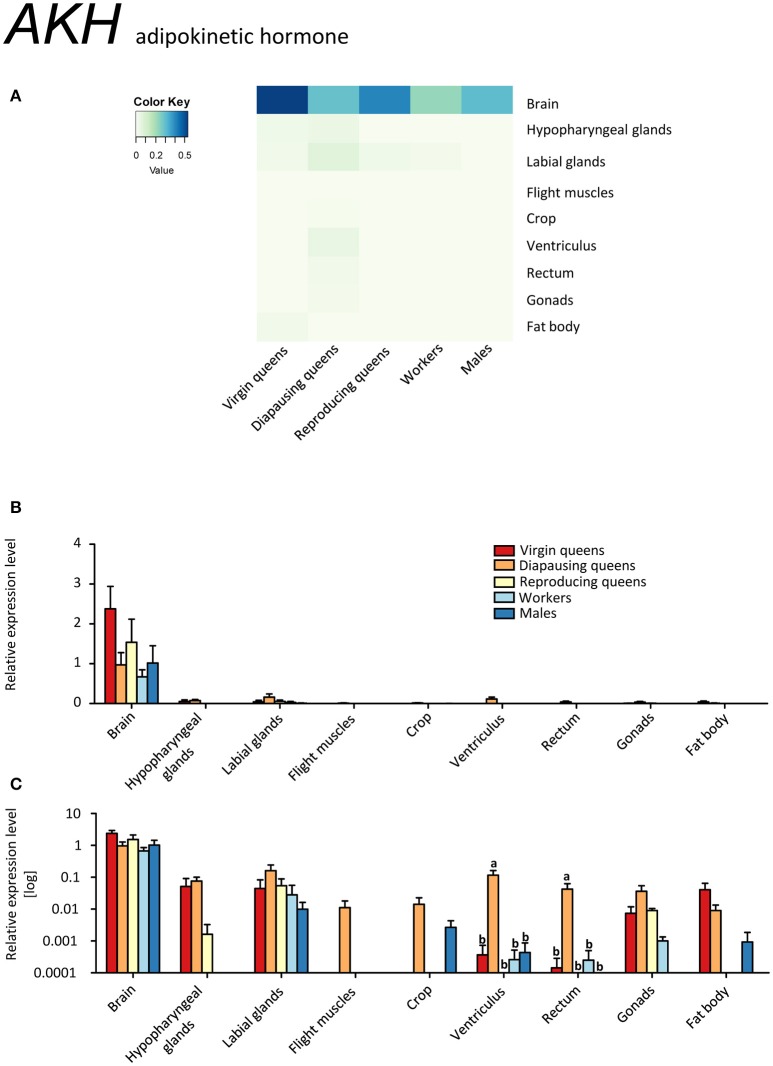
**qRT-PCR analysis of tissue-specific relative expression levels of *adipokinetic hormone* (*AKH*) across castes of *B. terrestris***. AKH is involved in the mobilization of lipid, carbohydrates and/or proline stores. *AKH* is mainly expressed in brain-CC-CA samples, but minor expression occurred also in other tissues (see main text). Data represent mean ± SEM (*n* = 3–5) of normalized and rescaled expression levels. **(A)** Heatmap: Log-scale. Bar graphs: Relative transcript levels in linear scale **(B)** and log-transformed scale **(C)**. Significantly different expression levels are indicated by different letters (*p* < 0.05).

*AKH* was expressed in brain samples, which included also CC and CA, of all investigated specimens (Figures 9A–C, Supplementary Table S2). Unexpectedly, a minor expression was also detected in other tissues. *AKH* expression outside of CC was previously reported in fat bodies of *A. mellifera* workers (Wang et al., 2012) and brain tissues (i.e., without CC-CA) of the red firebug, *P. apterus*, where the translated neuropeptide was also localized and quantified (Kodrík et al., 2015). However, the significance of these observations is not clear yet.

**Figure 10:**
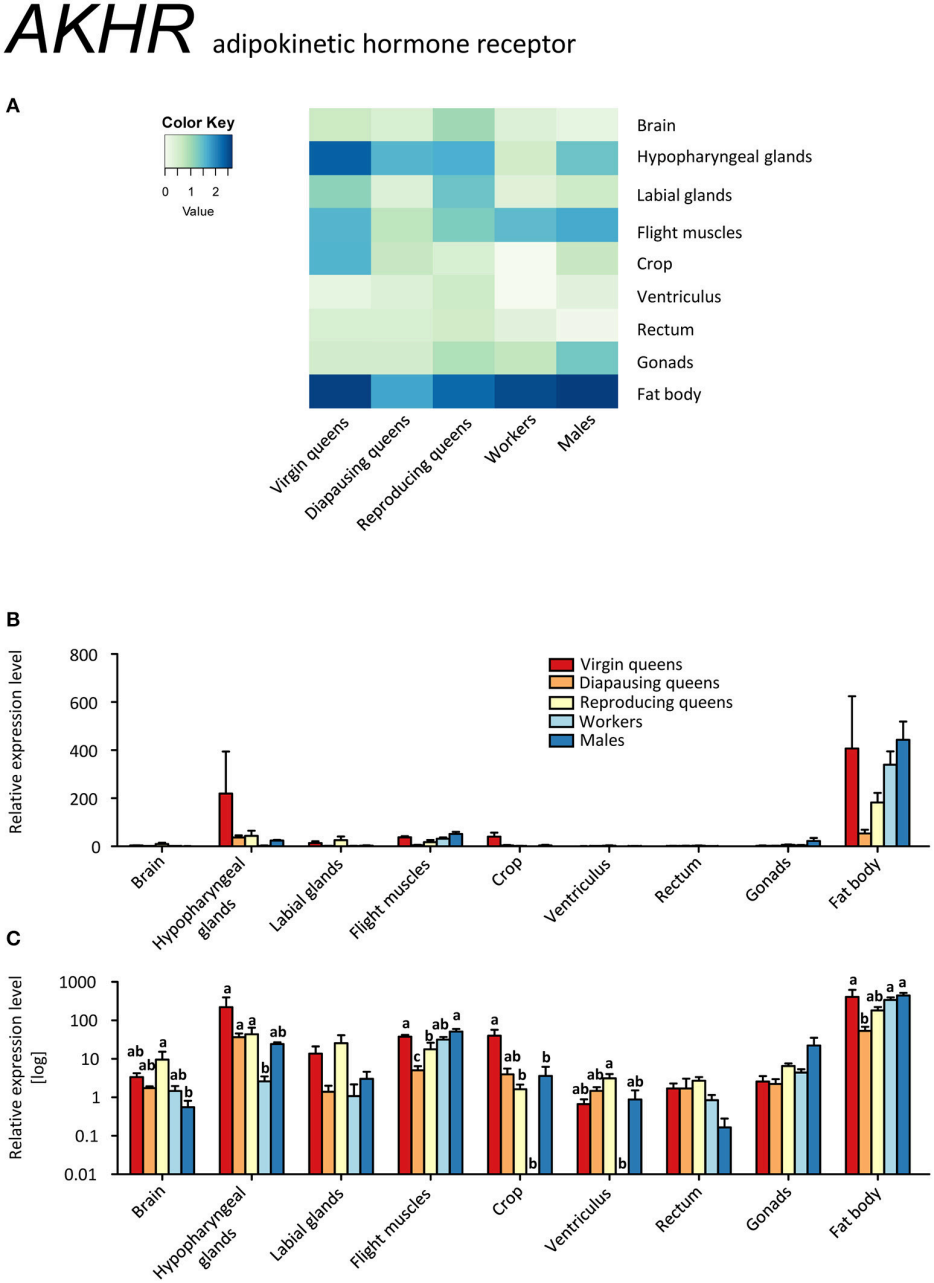
**qRT-PCR analysis of tissue-specific relative expression levels of *adipokinetic hormone receptor* (*AKHR*) across castes of *B. terrestris***. AKHR is involved in the regulation of energy storage. It is mainly expressed in fat body and hypopharyngeal glands (see main text). Data represent mean ± SEM (*n* = 3–5) of normalized and rescaled expression levels. **(A)** Heatmap: Log-scale. Bar graphs: relative transcript levels in linear scale **(B)** and log-transformed scale **(C)**. Significantly different expression levels are indicated by different letters (*p* < 0.05).

*AKHR* was ubiquitously expressed across all samples (Figures 10A–C, Supplementary Table S2). Its highest expression was observed in the fat body and flight muscles, as expected for a receptor involved in the release of carbohydrates and lipids upon high energy demand (Gäde and Auerswald, 2003; Gäde, 2009). Motionless, diapausing queens had the lowest levels, in agreement with their low energy demand. Surprisingly high expression levels of *AKHR* were recorded in hypopharyngeal glands, especially in virgin queens. As for other genes, *AKHR* expression in hypopharyngeal glands paralleled that of fat bodies. Whereas the presence of hypopharyngeal glands across bumblebee sexes and castes, as well as the synthesis of royal jelly-like protein within its cells, were reported recently (Albert et al., 2014), the overall significance of the glands remains unresolved. It was proposed that the original role of major royal jelly proteins was pre-digestive food modification, comparable to saliva (Kupke et al., 2012), and that the nutritive function seen in honeybees is derived. Similarly, the hypopharyngeal glands secretion contains digestive enzymes such as amylase and invertase (Pereboom, 2000). These reports together with our findings point to common mechanisms of endocrine regulation for food uptake and digestion as well as nutrient storage and release. Since AKH is actively involved in the control of digestive processes (Bodláková et al., 2016 and references therein), we propose that the high AKHR expression in hypopharyngeal glands is related to the regulation of digestive enzymes via AKH signaling.

Glycogen from skeletal muscles is considered to be one of the primary energy substrates for the initiation of insect flight (Beenakkers et al., 1984) due to AKH's capacity to activate glycogen phosphorylase (Gäde and Auerswald, 2003; Van der Horst, 2003). Indeed, the elevated expression of *AKHR* in the flight muscles of all active fliers (i.e., virgins, workers, and males; Figures 10A–C) supports this assumption.

### Queen's life cycle vs. AKH/AKHR signaling

Diapausing queens revealed the lowest *AKHR* expression, except for the digestive tract (Figures 10A–C, Supplementary Table S2). Moreover, the expression of *AKH* in the brain stays high over the course of the queen's life, and is higher than in workers and males. Studies on the regulation of *AKH* and *AKHR* gene expression in different phenotypes or in response to changes in the environment are limited to aphid models so far (Jedličková et al., 2015). The possible involvement of AKH/AKHR signaling in energy storage mobilization during diapause emerged with records of high sensitivity of diapausing *P. apterus* females to AKH application when compared to non-diapausing conspecifics (Socha and Kodrík, 1999). Here, we documented that AKH signaling in diapausing *B. terrestris* queens is downregulated via reduced *AKHR* expression. However, since the AKH of diapausing queens is expressed at similar levels in virgins and reproducing females, we cannot exclude that the significant depletion of glycogen during the course of hibernation reported by Amsalem et al. (2015b) may be attributed to the AKH/AKHR signaling.

## Conclusions

The virgin stage is characterized by the search for food and energy accumulation via upregulated FOXO, downregulated IIS and JH signaling and relatively high AKH/AKHR signaling. *AKH* expression is significantly higher than in other active fliers, i.e., workers and males, in line with the highest need for energy mobilization in virgin queens for active search of food prior to mating and hibernation. The *Kr-h1* expression pattern is opposite to that of *Vg* not only in virgins, but also in all other investigated phenotypes. Hence, it is unlikely that this transcription factor is directly involved in reproduction of bumblebees. The morphogenetic factor Kr-h1 is known to be engaged, among others, in cell division, taking place especially in ovaries of reproducing queens and workers and testes of males, and tissue remodeling in mated females, possibly present also in mated diapausing queens. Therefore, we hypothesize that the striking downregulation of *Kr-h1* expression in virgins might be connected to the absence of these processes.

Diapausing queens showed the expected downregulation of JH signaling, i.e., low *MFE* and *Vg* expression. *Kr-h1* occurred at surprisingly high abundances. We suggest that this may be due to tissue reconstruction in mated queens prior to upcoming reproduction. Queen-specific IGF-1 signaling is active in diapausing females as well. Thus, together with AKH/AKHR, IIS could be involved in co-regulation of energy turnover from the fat body storage during hibernation.

Reproductive status is unequivocally defined by upregulated JH signaling (*MFE* and *Vg*) and IIS, highlighted by suppressed *InR* genes probably due to highest values of *IGF-1* and *LIRP*. Specifically, IGF-1 was found to be a queen-specific insulin-like hormone. Thus, we propose it to play an essential role in the completion of egg development.

Workers showed the same regulation pattern of *MFE* and *Vg* as reproducing queens. By contrast, since *LIRP* is downregulated and *IGF-1* expression is even absent, the IIS is the most visible difference between workers and reproducing queens of *B. terrestris*. We hypothesize that arrested ovary development in workers could be connected to suppressed *IGF-1* expression, likely caused by the presence of the reproducing queen in the colony.

Males showed high values of *Kr-h1* and relatively low *Vg* expression. As in workers, IIS is mediated by both investigated InRs and one ligand, LIRP, but not by IGF-1.

When comparing the expression patterns of all studied genes in tissues dissected from all castes, we found striking similarities between the fat body and hypopharyngeal glands. Therefore, we suggest that hypopharyngeal glands, an organ presumably intervening in digestion immediately after food intake, are subjected to similar hormonal control mechanisms as the ultimate storage organ, the fat body.

## Author contributions

PJ, RH, and IV designed the study; PJ, UE, and AV acquired and analyzed data. PJ and UE drafted the manuscript, and all authors revised it, approved the final version, and agree to be accountable for the content of the work.

### Conflict of interest statement

PJ, UE, RH, and IV declare that the research was conducted in the absence of any commercial or financial relationships that could be construed as a potential conflict of interest. AV is an employee of Agricultural Research, Ltd., Troubsko, Czechia, a company that, among other activities, produces bumblebee colonies for pollination purposes. The reviewer DD declared a shared affiliation, though no other collaboration, with several of the authors PJ, UE, RH, IV to the handling Editor, who ensured that the process nevertheless met the standards of a fair and objective review.

## Supplementary material

Supplementary Table S1 **qRT-PCR primers and efficiencies used in this study**.

Supplementary Table S2 **ANOVA results for the ten genes investigated in this study**.

Supplementary Figure 1 **Example of tissue samples used in the present study**. The organs originated from a *B. terrestris* worker and represent: **(A)** Brain; **(B)** Hypopharyngeal glands; **(C)** Labial glands; **(D)** Flight muscles; **(E)** Gut: Crop (C), Ventriculus (V) and Rectum (R); **(F)** Gonads (Ovaries); and **(G)** Fat body.

Supplementary Figure 2 **The sequence analysis of insulin like peptide precursors. (A)** The alignment of *Bombus terrestris* IGF-1 (XP_012166281.1) and LIRP (XP_003400778.1) with related sequences of *Apis mellifera* (ILP-1: XP_016769293.1; ILP-2:NP_001171374.1 - incomplete sequence with RefSeq status: PROVISIONAL) and *Apis florea* (IGF-1: XP_012338912.1; LIRP: XP_003690101.1). The human insulin growth factor (*Homo sapiens* IGF-1a: NP_000609.1) and *Locusta migratoria* LIRP (P15131.2) are included as original references. Conserved residues are boxed in black, identical sequences in dark gray, and similar sequences in light gray. **(B)** The percent sequence identities of all the aligned peptides are presented.

## Abbreviations

AKH: adipokinetic hormone
AKHR: AKH receptor
CA: corpora allata
CC: corpora cardiac
Cp: crossing point
EF1: elongation factor eEF-1 α
FOXO: forkhead box protein O
IGF-1: insulin growth factor 1
IIS: insulin/insulin-like growth factor signaling
ILP: insulin-like peptide
InR: insulin-like receptor
InR-1: insulin-like peptide receptor 1
InR-2: insulin-like peptide receptor 2
JH: juvenile hormone
JHAMT: juvenile hormone acid methyltransferase
Kr-h1: Krüppel homolog 1
LIRP: locust insulin-related peptide
MFE: methyl farnesoate epoxidase
PLA2: phospholipase A2
TOR: target of rapamycin
Vg: vitellogenin.
