# Supplementary material for: Gene Expression Dynamics in Major Endocrine Regulatory Pathways along the Transition from Solitary to Social Life in a Bumblebee, Bombus terrestris
Source: Front Physiol. 2016 Nov 24;7:574. doi: 10.3389/fphys.2016.00574 (PMC5121236; doi:10.3389/fphys.2016.00574)
Supplement: Supplementary Figure 1 — Example of tissue samples used in the present study. The organs originated from a B. terrestris worker and represent: (A) Brain; (B) Hypopharyngeal glands; (C) Labial glands; (D) Flight muscles; (E) Gut: Crop (C), Ventriculus (V) and Rectum (R); (F) Gonads (Ovaries); and (G) Fat body. [file Image1.PDF]

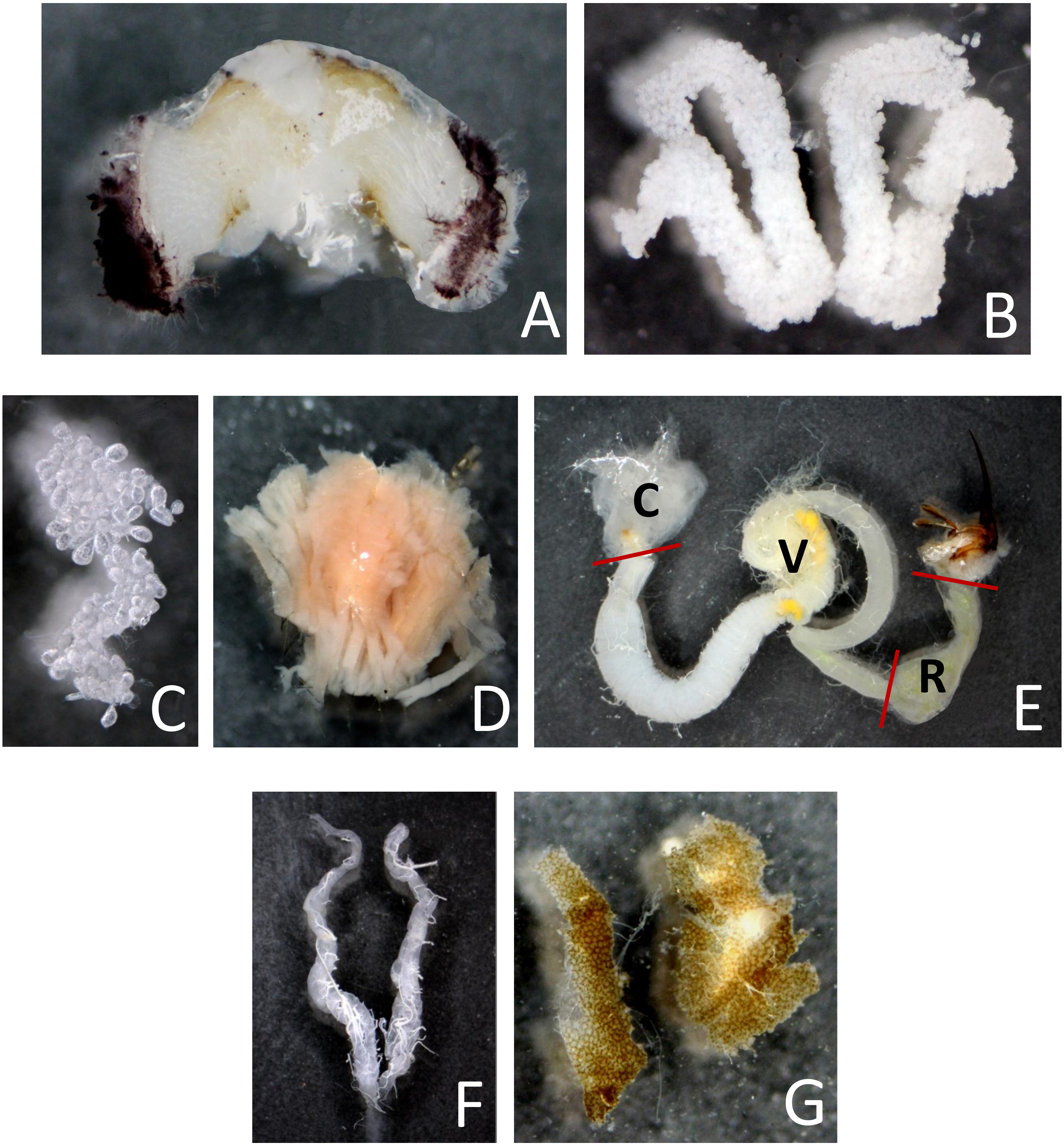

Supplementary Figure 1: Example of tissue samples used in the present study. The organs originated from a *B. terrestris* worker and represent: (**A**) Brain; (**B**) Hypopharyngeal glands; (**C**) Labial glands; (**D**) Flight muscles; (**E**) Gut: Crop (C), Ventriculus (V) and Rectum (R); (**F**) Gonads (Ovaries); and (**G**) Fat body.
