# Supplementary material for: Gene Expression Dynamics in Major Endocrine Regulatory Pathways along the Transition from Solitary to Social Life in a Bumblebee, Bombus terrestris
Source: Front Physiol. 2016 Nov 24;7:574. doi: 10.3389/fphys.2016.00574 (PMC5121236; doi:10.3389/fphys.2016.00574)
Supplement: Supplementary Figure 2 — The sequence analysis of insulin like peptide precursors. (A) The alignment of Bombus terrestris IGF-1 (XP_012166281.1) and LIRP (XP_003400778.1) with related sequences of Apis mellifera (ILP-1: XP_016769293.1; ILP-2:NP_001171374.1 - incomplete sequence with RefSeq status: PROVISIONAL) and Apis florea (IGF-1: XP_012338912.1; LIRP: XP_003690101.1). The human insulin growth factor (Homo sapiens IGF-1a: NP_000609.1) and Locusta migratoria LIRP (P15131.2) are included as original references. Conserved residues are boxed in black, identical sequences in dark gray, and similar sequences in light gray. (B) The percent sequence identities of all the aligned peptides are presented. [file Image2.PDF]

A

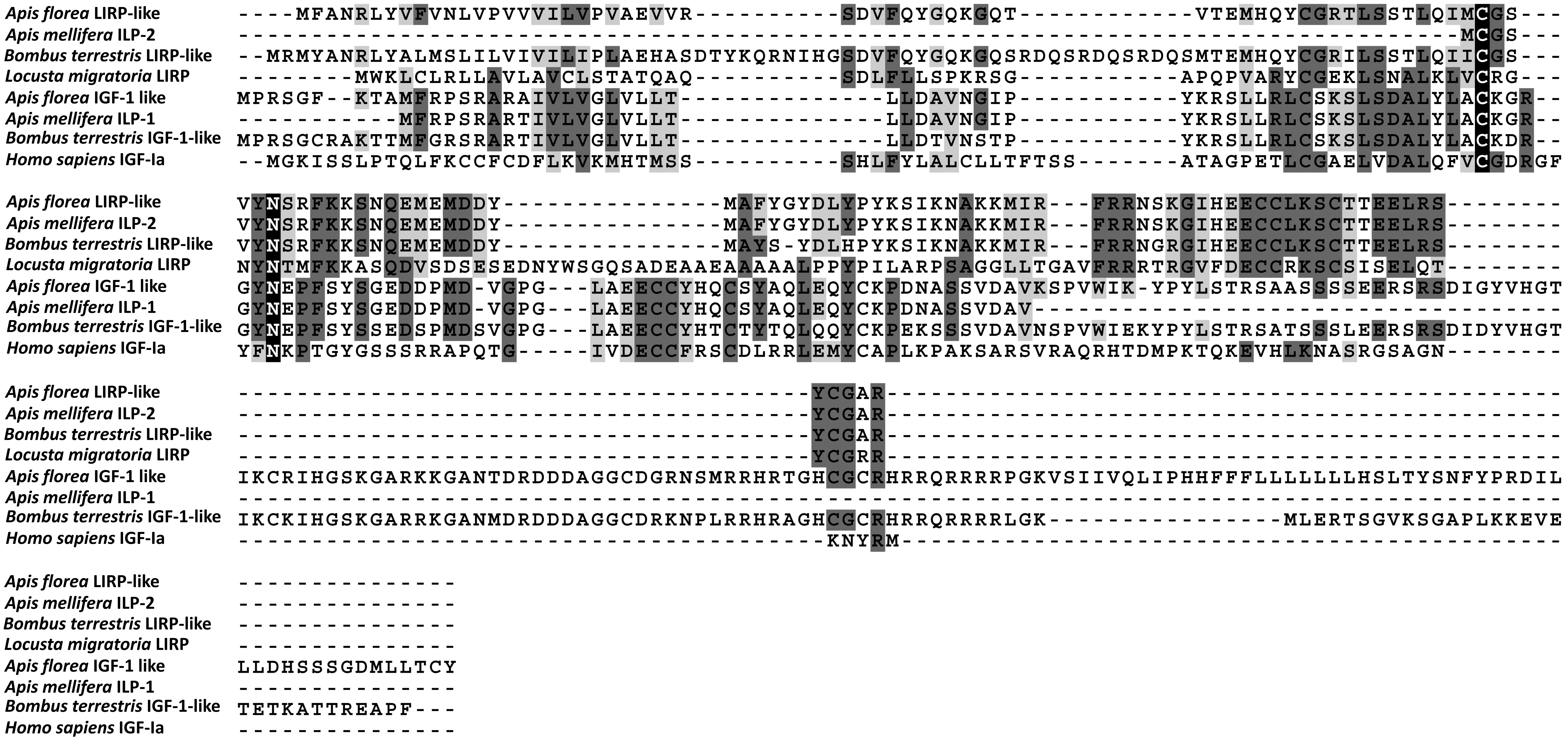

B

	B. terrestris	
	IGF-1	LIRP
A. mellifera ILP-1	36.9	18.5
A. mellifera ILP-2	11.0	44.2
A. florea IGF-1	67.1	13.1
A. florea LIRP	13.4	70.1
L. migratoria LIRP	18.2	28.5
H. sapiens IGF-1a	18.0	16.5

Supplementary Figure 2: The sequence analysis of insulin like peptide precursors. (A) The alignment of *Bombus terrestris* IGF-1 (XP_012166281.1) and LIRP (XP_003400778.1) with related sequences of *Apis mellifera* (ILP-1: XP_016769293.1; ILP-2:NP_001171374.1 - incomplete sequence with RefSeq status: PROVISIONAL) and *Apis florea* (IGF-1: XP_012338912.1; LIRP: XP_003690101.1). Further, the human insulin growth factor (*Homo sapiens* IGF-1a: NP_000609.1) and Locusta insulin related peptide (*Locusta migratoria* LIRP: P15131.2) are included as original references. Conserved residues are boxed in black, identical sequences in dark gray, and similar sequences in light gray. (B) The percent sequence identities of all the aligned peptides are presented.
